# Choroid Segmentation of Retinal OCT Images Based on CNN Classifier and *l*_2_-*l*_*q*_ Fitter

**DOI:** 10.1155/2021/8882801

**Published:** 2021-01-15

**Authors:** Fang He, Rachel Ka Man Chun, Zicheng Qiu, Shijie Yu, Yun Shi, Chi Ho To, Xiaojun Chen

**Affiliations:** ^1^Department of Applied Mathematics, The Hong Kong Polytechnic University, Hong Kong; ^2^Laboratory of Experimental Optometry, Centre for Myopia Research, School of Optometry, The Hong Kong Polytechnic University, Hong Kong; ^3^Blue Balloon Innovative Limited, Hong Kong; ^4^Centre for Eye and Vision Research, Hong Kong

## Abstract

Optical coherence tomography (OCT) is a noninvasive cross-sectional imaging technology used to examine the retinal structure and pathology of the eye. Evaluating the thickness of the choroid using OCT images is of great interests for clinicians and researchers to monitor the choroidal thickness in many ocular diseases for diagnosis and management. However, manual segmentation and thickness profiling of choroid are time-consuming which lead to low efficiency in analyzing a large quantity of OCT images for swift treatment of patients. In this paper, an automatic segmentation approach based on convolutional neural network (CNN) classifier and *l*_2_-*l*_*q*_ (0 < *q* < 1) fitter is presented to identify boundaries of the choroid and to generate thickness profile of the choroid from retinal OCT images. The method of detecting inner choroidal surface is motivated by its biological characteristics after light reflection, while the outer chorioscleral interface segmentation is transferred into a classification and fitting problem. The proposed method is tested in a data set of clinically obtained retinal OCT images with ground-truth marked by clinicians. Our numerical results demonstrate the effectiveness of the proposed approach to achieve stable and clinically accurate autosegmentation of the choroid.

## 1. Introduction

Choroid is the vascular layer located between retina and sclera. Its inner surface is connected with the retinal pigment epithelium (RPE) through Bruch's membrane (BM), and the outer surface is connected with the sclera. Recent researches indicated that the changes of the choroidal thickness could be related to some ocular conditions such as macular degeneration and myopia [1–4]. Therefore, segmentation and the ability to accurately measure the thickness of the choroid are of clinical importance.

Optical coherence tomography (OCT) is a technique for obtaining subsurface images of translucent or opaque materials with high resolution [5, 6]. It uses low-coherence interferometry and imaging reflections from interior tissues to generate cross-sectional images. Comparing with traditional imaging methods, OCT has some obvious advantages of being nondestructive, high resolution, and minimally invasive, and it has been widely applied in ocular detections for many years [3, 7, 8]. However, the imaging quality of the choroid is not good enough in retinal OCT images due to the shortage of penetration depth [9]. The major challenges of the choroidal segmentation are from low contrast of the lower boundary and unknown noise in the images, which will make the detection result inaccurate and unreliable.

To segment the choroid efficiently, many researchers studied model-based methods with prior assumptions for the structure of the input images: A-scan [10, 11], active contour [12–15], sparse high order potentials [16], and 2D/3D graph [17–22] methods. The main limitation of these traditional approaches is their high dependence on the feature extraction phase for the accurate segmentation. However, extraction of appropriate image features is difficult for a definite medical image recognition problem, and the traditional methods may provide disappointing segmentation results. In recent years, machine learning-based methods have achieved excellent performance in computer vision and medical image analysis. Convolutional neural network (CNN) is one of the extensive application approaches for image processing and also effective for multilayer segmentation in OCT images. Sui et al. used a graph-searching-based segmentation technique with learning an optimal graph weight by using CNN architecture [23]. Masood et al. proposed a two-stage segmentation method to segment out the BM and choroid and calculate the thickness map [24]. A series of morphological operations were used to segment BM, while the choroid was segmented using CNN. Fang et al. presented a novel framework combining CNN and graph search methods (CNN-GS) to segment nine layer boundaries on retinal OCT images [25]. Alonso-Caneiro et al. used a fully-convolutional network (FCN) technique based on graph search theory to segment the choroidal boundary and obtain the choroidal thickness profile from OCT images [26]. There are also some other outstanding networks for OCT image segmentation such as U-shape convolutional network (U-Net), which is considered as the most widely applicable architecture for medical image segmentation [27, 28].

It is worth noting that a major disadvantage of many machine learning-based methods, such as CNN-GS, FCN-GS, and U-net, is their reliance on the availability of a large supervised/marked data set. However, marking medical images manually requires highly professional technique, which leads to the lack of accurately labeled data in great quantity. In addition, the number of negative samples is far more than the number of positive samples, i.e., the pixels within a single OCT image are largely labeled as “0” (not contained in the Choroidal-Scleral interface boundary) as opposed to being positively labeled as “1” (contained in the Choroidal-Scleral interface boundary), which may affect the training results. In extreme cases, the loss of negative samples will dominate in the training process and may lead to a high accuracy even when the model predicts all the samples to be negative. Taking these into considerations, we propose an improved CNN model-based method which performs well on a small data set and reduces the adverse impact of unbalanced samples. With the choroid boundaries obtained by neural network, we further adopt a *l*_2_-*l*_*q*_ (0 < *q* < 1) regression model to fit the choroidal layer curve. This model ensures not only the fitting accuracy but also the simplicity of fitting function, leading to a better generalization segmentation result.

This paper is organized as follows. In Section 2, we describe the details of the proposed method including segmentations of the BM and Choroidal-Scleral interface. Experimental evaluation and comparison with other methods are discussed in Section 3. Concluding remarks are given in Section 4.

## 2. Materials and Methods

### 2.1. OCT Data

OCT images were obtained in Chinese schoolchildren aged 8-13 years using spectral domain OCT (SD-OCT, Spectralis HRA+OCT, Heidelberg Engineering, Germany) in Optometry Research Clinic of The Hong Kong Polytechnic University. Consents were obtained from both children and their parents/guardians. The study protocol has been approved by the Human Subjects Ethics Subcommittee of The Hong Kong Polytechnic University and met the tenets of the Declaration of Helsinki. Cross-sectional OCT images with axial resolution of 3.9 *μ*m and transverse resolution of 14 *μ*m were obtained using a light source (peak wavelength of 870 nm) together with a scanning speed of 40000 A-scan/sec. In order to better capture the boundary of choroid, an enhanced depth image scanning mode was adopted. Choroid was then manually segmented using the built-in software in SD-OCT by trained clinicians. There are 146 marked retinal OCT images from 108 patients in total, and we divide them into a training set of 70 images, while the rest are categorized as in the testing set. In our experiments, all the OCT images are preprocessed as grey images with the size of 150 × 600 (height × width) pixels.

### 2.2. Overview

**Figure 1:**
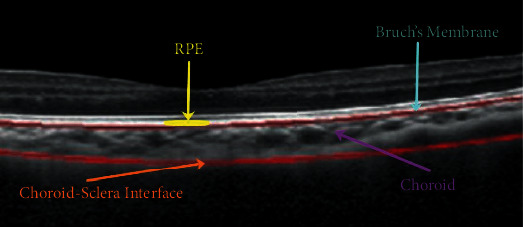
Physiological structure of BM, RPE, choroid, and Choroidal-Scleral interface. Red curves are the ground-truth marked by clinicians.

The proposed approach is divided into two parts: BM segmentation and Choroidal-Scleral interface segmentation. Physiological tissues mentioned in this work are visualized by diverse colors in Figure 1, and red curves mark the known ground-truth provided by clinicians. For BM segmentation, we start by recognizing the approximate position of RPE, which is the brightest layer in retinal OCT images. We then identify the BM by its physiological feature in the vicinity of RPE. After the extraction of the BM, we consider to segment the Choroidal-Scleral interface by a two-stage process:

1. Partition the OCT image into small patches and input them into the CNN based classifier. The likelihood of the Choroidal-Scleral interface passing through the patch increases with the predicted value's proximity to 1.
2. Generate a heat map according to the predicted values of patches. Choose appropriate points in the heat map and fit these points to obtain the curve of the Choroidal-Scleral interface.

The details will be discussed in the later sections.

Our numerical experiments in this paper are implemented in Tensorflow 1.13.1, Python 3.6.6, and Cuda 9.0, running on a server with 2 Tesla P100-PCIE GPU with 16 GB memory at 1.3285 GHz and an operating system of 64 bits in the University Research Facility in Big Data Analytics (UBDA) of the Hong Kong Polytechnic University. (UBDA website: https://www.polyu.edu.hk/ubda/.)

### 2.3. Retinal Pigment Epithelium Recognition

Since RPE is the brightest part in all retinal OCT images after the reflection of light, we can locate the approximate position of RPE. Regarding the OCT image as a 150 × 600 matrix, the point with the largest pixel value in each column can be found and denoted by *P*_*i*_, *i* = 1, ⋯, 600. Fitting the points {*P*_*i*_, *i* = 1, ⋯, 600} and then we can obtain a curve *C*_*RPE*_ lying in the region of RPE. Here, we adopt the cubic regression method in consideration of the simplicity of the RPE curve and the high accuracy of extracted points.

### 2.4. Bruch's Membrane Segmentation

**Figure 2:**
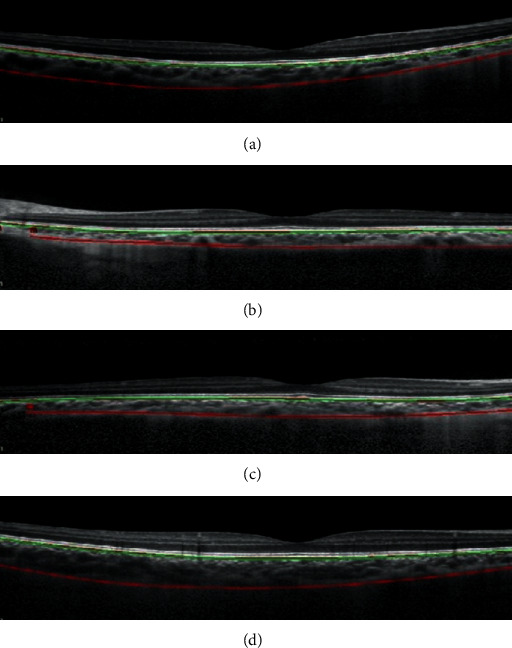
Green curves are BM segmentation results, red curves are ground-truth of BM and Choroidal-Scleral interface marked by clinicians.

From Figure 1, we can observe that the BM is at the boundary between RPE and the choroid, and below the curve *C*_*RPE*_ extracted in the previous step. Therefore, we intend to find appropriate points *Q*_*i*_, *i* = 1, ⋯, 600 in the lower neighborhood of *C*_*RPE*_, where *Q*_*i*_ is with the largest difference in magnitude in the *i*th column. In our experiments, the set {*Q*_*i*_, *i* = 1, ⋯, 600} is obtained in the region between *C*_*RPE*_ and *C*_*RPE*−5_; *C*_*RPE*−5_ represents the curve whose point in each column is 5 pixels lower than the corresponding point in *C*_*RPE*_. The width of gap between two curves is decided by the observation that the thickness of RPE is about 5 pixels in OCT images. Finally, the curve acquired by fitting points {*Q*_*i*_, *i* = 1, ⋯, 600} is regarded as the BM. Some results of BM segmentation are shown in Figure 2; we can find that our result (green curve) and ground-truth of BM (red curve) coincide with each other. The error for each image is calculated by

(1) $$\begin{array}{c} \frac{1}{600}\overset{600}{\underset{i=1}{\sum }}\left|\text{BM}\left(i\right)-\hat{\text{BM}}\left(i\right)\right|, \end{array}$$

where BM(*i*) and $\hat{\text{BM}}\left(i\right)$ represent the corresponding numbers of row for *i*th column in our result and ground-truth of BM, respectively. The average error and variance for 76 test images are 1.5189 and 1.1325.

### 2.5. Choroidal-Scleral Interface Segmentation

In this section, we present the details of the method based on CNN classifier and *l*_2_-*l*_*q*_ (0 < *q* < 1) fitter to segment the Choroidal-Scleral interface.

#### 2.5.1. Data Preprocessing and Clipping

In order to improve the recognition accuracy of the lower choroidal boundary, we first remove the irrelevant information in the OCT images above the BM. A large size of patch may lead to a small number of samples, while a small size of patch will result in the lack of features in samples. In our proposed approach, we cut images from the training set into a group of square patches with 32 × 32 pixels from left to right, top to bottom with a step-size of 8.

**Figure 3:**
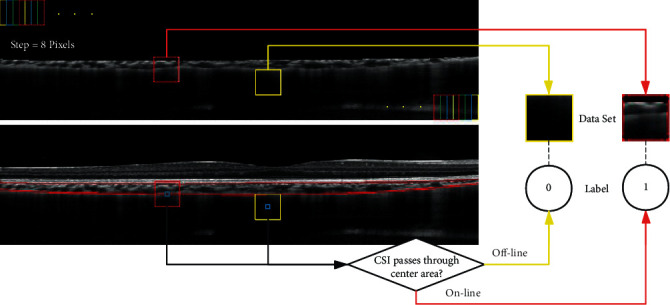
Red curve: Ground-truth. Yellow minipatch: off-line patch with label “0.” Red minipatch: on-line patch with label “1.” Blue center-patch: recognition area with size of 6 × 6.

For each 32 × 32 minipatch, if the marked ground-truth passes through its 6 × 6 center area, this minipatch will be labeled as “1” (positive sample) and as “0” (negative sample) otherwise. As shown in Figure 3, the minipatch in red is labeled as “1,” and the minipatch in yellow is labeled as “0.” The blue frame in the center is the recognition area with a size of 6 × 6 pixels. In [24], the criterion to define positive samples is the marked ground-truth passes through the 32 × 32 patch, otherwise is a negative sample. Thus, the number of positive samples made in [24] is more than ours, and the ratio of positive and negative samples is relatively balanced. However, the positive samples whose edges are passed through by the marked ground-truth may impact the extraction of features in training process. Therefore, we set the 6 × 6 recognition area in our samples to avoid this problem. After finishing the labeling, the layer segmentation problem is converted into a binary classification problem.

#### 2.5.2. CNN Training

The structure of the neural network used in our work is the Lenet-5 model [29]. It includes 3 convolutional layers and 3 fully connected layers, which is shown in Table 1. Each convolutional layer consists of a layer of convolution and function of local response normalization. The purpose of 3 continuous convolutional layers is to extract features and map the original data into a feature space. The kernel size in each convolution layer is 3 × 3. After the process of feature extraction, 3 fully connected layers are arranged to provide a classification.

Before applying this CNN model to segment the choroid, it is necessary to pretrain it by using training data. After data preprocessing and clipping, we can get 260000 patches with label as a revised training set. Among these patches, about 24000 samples are on-line samples with label 1, while the other 236000 patches are off-line samples with label 0. Note that the ratio of positive and negative samples is about 1 : 11 due to the way of making samples and specialty of images. As mentioned earlier, unbalanced samples will have a negative effect on the training result, and it is necessary to provide a reasonable solution. For clear display, we use *f*_CNN_^*θ*^ : ℝ^32×32^ → (0, 1) to represent the CNN model; then, the relationship between the input patches *img*_*i*_ and the output values *p*_*i*_ can be denoted as

(2) $$\begin{array}{c} p_{i}=f_{\text{CNN}}^{\theta }\left(img_{i}\right), \end{array}$$

where *img*_*i*_ is the *i*th patch of our input and *θ* is the set of parameters in the CNN model. The problem of unbalanced samples, the ratio of on-line samples, and off-line samples is about 1 : 11 and is treated by our adoption of a loss function called Focal Loss [30]:

(3) $$\begin{array}{c} \begin{array}{r} \begin{array}{rr} & \text{FL}\left(p_{i},l_{i}\right)\\ & =-\alpha l_{i}{\left(1-p_{i}\right)}^{\gamma }\ln\left(p_{i}\right)-\left(1-\alpha \right)\left(1-l_{i}\right)p_{i}^{\gamma }\ln\left(1-p_{i}\right), \end{array} \end{array} \end{array}$$

where *l*_*i*_ is the label of the corresponding patch, *α* ∈ (0, 1) ensures a higher weight for the rare online samples, and *γ* ≥ 0 provides a higher weight for samples hard to be classified.

The Focal Loss, a dynamically scaled cross-entropy loss function, is proposed for dealing with samples' imbalance in [30]. From formula (3), we can find that the value of loss function is reducing with a higher *p*_*i*_, which means the class with lower accuracy will dominate in network training.

Next, we need to minimize FL(*p*_*i*_, *l*_*i*_). Let *g*_*i*_(*θ*) = *f*_CNN_^*θ*^(*img*_*i*_), and the problem can be formulated as

(4) $$\begin{array}{c} \begin{array}{r} \arg\underset{\theta }{\min}\;\frac{1}{I}\overset{I}{\underset{i=1}{\sum }}\text{F}\text{L}\left(g_{i}\left(\theta \right),l_{i}\right), \end{array} \end{array}$$

where *I* denotes the total number of input patches. To prevent overfitting in CNN, Dropout and *l*_2_ regularization have been used [31–33]. In our numerical experiments, we add *l*_2_ regularization in the objective function to prevent overfitting; hence, the regularized problem is written as

(5) $$\begin{array}{c} \begin{array}{r} \arg\underset{\theta }{\min}\;J\left(\theta \right)≔\frac{1}{I}\overset{I}{\underset{i=1}{\sum }}\text{F}\text{L}\left(g_{i}\left(\theta \right),l_{i}\right)+\frac{\mu }{2}||\theta {||}_{2}^{2}. \end{array} \end{array}$$

Adaptive Moment Estimation (Adam) optimizer [34] is applied to solve problem (5).

**Figure 4:**
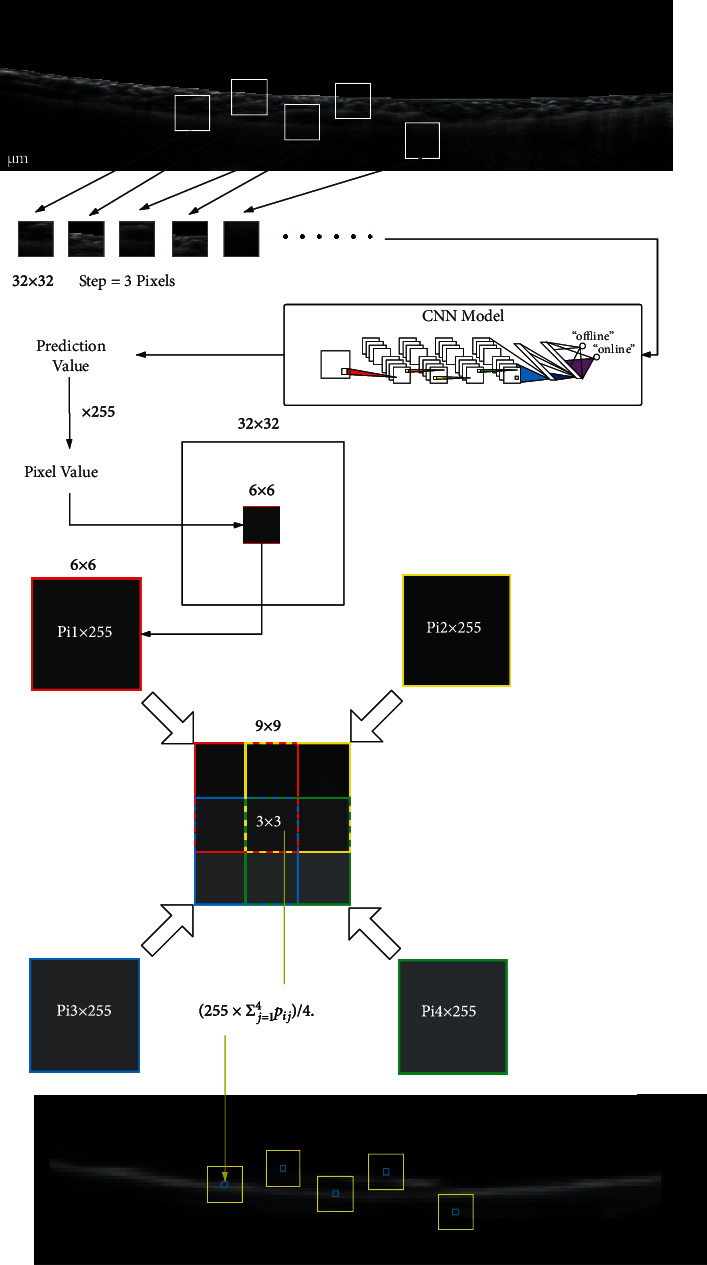
An example for the calculation of element value in *M*.

After finishing the training of the CNN model, we then apply it to segment choroid. Let *M* ∈ ℝ^150×600^ be a matrix for the storage of output values corresponding to the image matrix. At the first stage, we need to implement the following operations:

1. Cut the input OCT image into a group of patches with size of 32 × 32 from left to right, top to bottom with a step-size 3. Every patch matches a submatrix of *M* with a size of 6 × 6,
2. Input these patches into the trained CNN model one by one and obtain the corresponding predicted value, denoted by *p*_*i*_ for the *i*th patch, *i* = 1, ⋯, 7600. All the values of elements in the corresponding submatrix are *p*_*i*_,
3. The element value of *M* corresponding to the *i*th patch is replaced by 255 × *p*_*i*_. The element value of *M* in the overlaps of two or four patches is replaced by a weighted average value. For example in Figure 4, if it lies on the overlaps of four patches: the *i*_*j*_th patch for *j* = 1, 2, 3, 4, its element value is (255 × ∑_*j*=1_^4^*p*_*i*_*j*__)/4,
4. Find the second largest element (i.e., the center element of three continuous largest elements) in each column of matrix *M* as the desired point.

**Figure 5:**
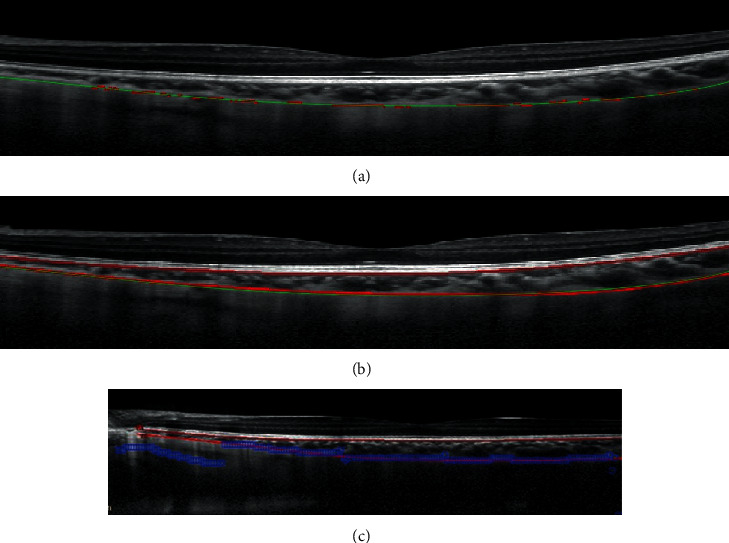
(a) The original image. (b) The image decoded by matrix *M*. (c) The vertical brightest area in each column of matrix *M*.

The results of the above process are shown in Figure 5, which is a special example with a lot of noises and mistaken points. Hence, we need to further recorrect the data.

#### 2.5.3. Data Recorrection

From the above results, we notice that the major area of the choroid can be accurately detected. However, there may be some misjudgments because of the interference information in the image, and these errors will make a negative influence on the segmentation result if we directly fit all the sample points without filter. For this reason, the random sample consensus (RANSAC) algorithm [35] is applied to recorrect the detected points. RANSAC algorithm estimates parameters of a mathematical model from a set of observed data that contains “outliers” by iterations. Basic assumptions of RANSAC are in the following:

- Data consist of “inliers”, i.e., the distribution of data can be explained by some set of model parameters.
- “Outliers” do not fit the model.
- Other data are regarded as noise data.

For RANSAC, it is also assumed that there exists a process of estimating model parameters for a set of given “inliers,” and this model can optimally explain or fit the data.

In addition, a shape constraint rule is enforced to help identifying the outlines, namely, the location of the choroid is always below the BM.

#### 2.5.4. *l*_2_ − *l*_*q*_ Fitter

This step sketches out the outer boundary of the choroid with recorrected data. Through the observation of the ground truth, a high-order polynomial *g*(*x*) = *β*_*n*_*x*^*n*^ + *β*_*n*−1_*x*^*n*−1^ ⋯ +*β*_1_*x* + *β*_0_ is applied for curve fitting to improve accuracy. Moreover, to avoid the fitting function being too complicated for stability, we preferably set a relatively sparse group of coefficients {*β*_*n*_, ⋯, *β*_1_, *β*_0_}. In recent years, *l*_*q*_ (0 < *q* < 1) regularization attaches attention and has advantages over smooth, convex regularization for variable selection. Motivated by this, a regression model consists of a *l*_2_ data fitting term and a *l*_*q*_ regularization term is used to ensure the sparsity of polynomial coefficients in this paper. The *l*_2_ − *l*_*q*_ regression model is as follows:

(6) $$\begin{array}{c} \begin{array}{r} \underset{\beta }{\min}\;\phi \left(\beta \right)≔||X\beta -y{||}_{2}^{2}+\lambda ||\beta {||}_{q}^{q}, \end{array} \end{array}$$

where

(7) $$\begin{array}{c} \begin{array}{r} X≔\left(\begin{array}{cccc} x_{1}^{n} & \cdots & x_{1}^{1} & 1\\ x_{2}^{n} & \cdots & x_{2}^{1} & 1\\ \vdots & \ddots & \vdots & 1\\ x_{m}^{n} & \cdots & x_{m}^{1} & 1 \end{array}\right),y≔\left(\begin{array}{c} y_{1}\\ y_{2}\\ \vdots \\ y_{m} \end{array}\right), \end{array} \end{array}$$

{(*x*_*i*_, *y*_*i*_), *i* = 1, ⋯, *m*} (*m* ≤ 600) denotes the set of recorrected coordinate points, *β* = (*β*_*n*_, *β*_*n*−1_,⋯,*β*_1_, *β*_0_)^*T*^ ∈ ℝ^*n*+1^ is the vector consists of the fitting polynomial coefficients, ||*β*||_*q*_^*q*^ = ∑_*i*=0_^*n*^|*β*_*i*_|^*q*^, and *λ* > 0 is a parameter.

Some existing methods such as iteratively reweighted *l*_1_ (IRL1) and *l*_2_ (IRL2) minimization algorithms have been widely studied for solving problem (6), see [36–39] and references therein. We adopt the hybrid orthogonal matching pursuit-smoothing gradient (OMP-SG) based on lower bound theory in [36] to solve problem (6). First, we use the OMP method to generate an initial point *β*^0^ and its support set; then, the SG method is employed to further reduce the objective value of (6), and finally, the numerical solution is purified by deleting its entries with small values. The framework of the hybrid OMP-SG algorithm is given in Algorithm 1, where |*Λ*| denotes the number of elements in the set *Λ*.

We refer the interested readers to [36] for more details on the OMP-SG algorithm. Moreover, the smoothing function used in the SG method is

(8) $$\begin{array}{c} \tilde{\phi }\left(\beta \right)={\left\|X\beta -y\right\|}_{2}^{2}+\lambda \overset{n}{\underset{i=0}{\sum }}{\left(s_{\xi }\left(\beta_{i}\right)\right)}^{q}, \end{array}$$

where

(9) $$\begin{array}{c} {\text{s}}_{\xi }\left(\text{t}\right)=\left\{\begin{array}{c} \left|t\right|,\left|t\right|>\xi \\ \frac{{\text{t}}^{2}}{2\xi }+\frac{\xi }{2},\left|t\right|\leq \xi \end{array}\right.. \end{array}$$

*ξ* > 0 is a smoothing parameter. It is easy to verify that $\tilde{\phi }$ is continuously differentiable for any fixed *ξ* > 0.

Since *ϕ*(*β*) ≥ *λ*‖*β*‖_*q*_^*q*^, the objective function *ϕ*(*β*) is bounded below and *ϕ*(*β*) → ∞ if ||*β*||_*q*_ → ∞. Moreover, the set of local minimizers of problem (6) is nonempty and bounded. According to Theorem 2.2 in [36], for any local minimizer *β*^∗^ of problem (6) satisfying *ϕ*(*β*^∗^) ≤ *ϕ*(*β*^0^), a lower bound theory of nonzero entries and an upper bound of ‖*β*^∗^‖_0_ = #{*β*_*i*_^∗^ ≠ 0, *i* = 0, 1, ⋯, *n*} are presented as follows.

**Theorem 1 .**

Let *β*^∗^ be a local minimizer of problem (6) satisfying *ϕ*(*β*^∗^) ≤ *ϕ*(*β*^0^) for an arbitrarily given point *β*^0^. Let $L={\left(\lambda q/2||X{||}_{2}\sqrt{\phi \left(\beta^{0}\right)}\right)}^{1/1-q}$; then, we have $\begin{array}{r} \beta_{i}^{*}\in \left(-L,L\right)\Rightarrow \beta_{i}^{*}=0,\text{for }i\in \left\{0,1,\cdots ,n\right\}. \end{array}$ Moreover, the number of nonzero entries in *β*^∗^ is bounded by ‖*β*^∗^‖_0_ ≤ min(*m*, (*ϕ*(*β*^0^)/*λL*^*q*^)).

From the upper bound in Theorem 1, the number of nonzero elements of *β*^∗^ is less than *ϕ*(*β*^*omp*^)/*λL*^*q*^. The sparsity of *β*^∗^ is dependent on the choice of *λ*. A sufficient condition on *λ* for minimizers of problem (6) to have desirable sparsity can be found in [40].

**Figure 6:**
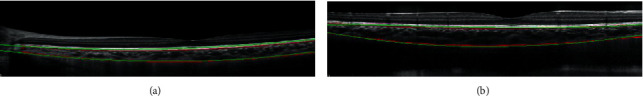
Curve fitting result by *l*_2_ − *l*_*q*_ regression model with *λ* = 1, *ϵ* = 0.1, *n* = 10, *q* = 0.1. (a) Red part is a scatter of recorrected points. Green part is the fitting result by the obtained high-order polynomial. (b) Red curves are ground-truth of BM and Choroidal-Scleral interface marked by a clinician. Green curve is fitting result.

**Figure 7:**
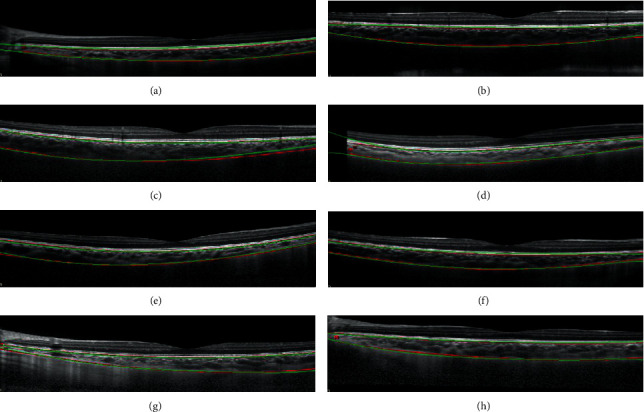
Green curve: segmentation results by the proposed method. Parameters are consistent with the parameters in Figure 6. Red curve: ground-truth.

With the output result by Algorithm 1, we can obtain a high-order polynomial whose coefficient vector is *β*^∗^ to fit the curve as shown in Figure 6(a). The red part is the scatter of recorrection points, i.e., {(*x*_*i*_, *y*_*i*_), *i* = 1, ⋯, *m*}, and the fitting curve is shown in green. In Figure 6(b), we compare the fitting result and ground-truth in green and red, respectively.  Figure 7 shows some of the choroid segmentation results with images in the testing set. These examples display that the regression fitting is visually reasonable and conforms to the ground-truth.

### 2.6. Summary

In our experiments, we set *λ* = 1, *q* = 0.1, *μ* = 0.1, *α* = 0.9, and *γ* = 2. For achieving a more stable fitting result, we choose the value of *n* in (6) according to the recorrection data {*x*_*i*_, *i* = 1, ⋯, *m*}(*m* ≤ 600) of the given image as following:

- *n* = 10, if *x*_1_ ≤ 50 and *x*_*m*_ ≥ 570,
- *n* = 3, if *x*_1_ ≥ 100 or *x*_*m*_ ≤ 500,
- *n* = 6, if the above two conditions are not satisfied.

**Figure 8:**
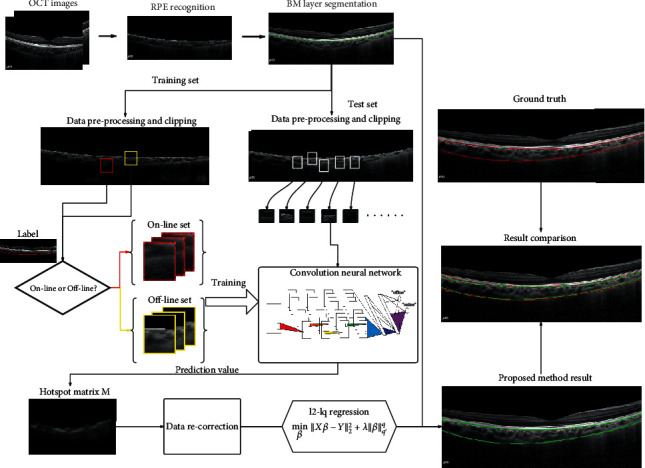
Summary of our proposed choroid segmentation method.

In Figure 8, we give a flowchart to show the process of our choroid segmentation method. The RPE and BM are recognized first by their physiological characteristics. The labeled patches cut from OCT images are used to train a CNN classifier. Then, the input image can be transferred into a group set of points with a high predicted value by the trained CNN model. After data recorrection by the RANSAC method, the residual points are fitted by a *l*_2_ − *l*_*q*_(0 < *q* < 1) regression model, and the fitting curve is the desired outer interface of the choroid. Thus, we obtain the segmentation results of the choroid.

## 3. Results and Discussion

In this section, we discuss the experimental analysis based on thickness evaluation. We give the measurement criteria of thickness, and then compare the average error, maximal error, and Dice coefficient of our experimental results with the results by the method in [24].

### 3.1. F Hypothesis Testing and T Hypothesis Testing

We use the Anderson-Darling test to verify both the average thickness marked by clinicians and predicted by our model with approximate normal distribution. Therefore, we can adopt F Hypothesis testing and T Hypothesis testing to analyze the thickness results obtained by our method.

By F Hypothesis testing, we can compute the confidence level of the thickness results. Denote *X*^0^ = {*x*_*i*_^0^, *i* = 1, ⋯, *h*} by the thickness sample set in which the element represents an average thickness of choroid given by medical staff in one OCT image. *X*^1^ = {*x*_*i*_^1^, *i* = 1, ⋯, *h*} is the thickness sample set given by our proposed method. First, we use the *F*-test to verify whether the variances between the given thickness samples *X*_0_ and our thickness results *X*_1_ are similar.

- *H*_0_: there is no significant difference in the variance between the given sample and our result sample
- *H*_1_: there is a significant difference in the variance between the given sample and our result sample

Value of *F*-testing is

(10) $$\begin{array}{c} \begin{array}{r} F=\frac{S_{0}^{2}}{S_{1}^{2}}, \end{array} \end{array}$$

where *S*_0_ and *S*_1_ are the variances of samples in *X*^0^ and *X*^1^, respectively. The *P* value of *F*-test is 0.2092 > 0.05, i.e., *H*_0_ can not be rejected. In other words, it can be ensured that the results by our proposed method are not significantly different from the ground-truth.

After verifying the variance, the next step is to verify whether there is a significant difference between the mean values of the two sample sets.

- ${\bar{H}}_{0}$: there is no significant difference in the average value between the given sample and our result sample
- ${\bar{H}}_{1}$: there is a significant difference in the average value between the given sample and our result sample

Value of *T*-testing is

(11) $$\begin{array}{c} \begin{array}{r} T=\frac{{\bar{X}}^{0}-{\bar{X}}^{1}}{\sqrt{\sum {\left(x_{i}^{0}\right)}^{2}+\sum {\left(x_{i}^{1}\right)}^{2}/2h-2}\times \left(2/h\right)}, \end{array} \end{array}$$

where ${\bar{X}}^{0}$ and ${\bar{X}}^{1}$ are mean values of samples in *X*^0^ and *X*^1^, respectively. The *P* value of *T*-test is 0.2898 > 0.05, i.e., ${\bar{H}}_{0}$ can not be rejected. Therefore, the mean value of our results is not significantly different from the data given by optometrists.

### 3.2. Minimum Distance Method

**Figure 9:**
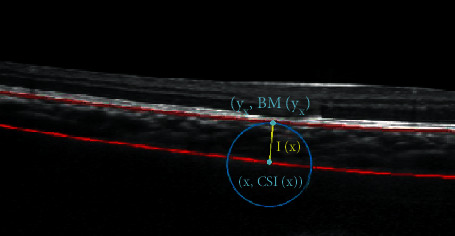
Minimum distance method.

In this part, we compare the results of our proposed method and other methods in detail. Since finding the minimum distance point directly in the image will produce a zigzag error, we calculate the distance based on the regression functions of the BM and choroid. Define the set of horizontal ordinate in the result image as *𝒮* = {*x*, 1 ≤ *x* ≤ 600}; CSI(*x*) and BM(*x*) represent the regression function value at *x*, respectively. The minimum distance method starts from a point (*x*, CSI(*x*)) on the choroidal curve, then finding the corresponding point (*y*, BM(*y*)) on the BM which is closest to point (*x*, BM(*x*)), and calculating the distance between these two points. As shown in Figure 9, the length of the yellow line is desired.

The thickness function *l*(*x*) at point *x* ∈ *𝒮* is defined as follows:

(12) $$\begin{array}{c} \begin{array}{r} \begin{array}{rr} l\left(x\right) & ≔||\left(x,\text{CSI}\left(x\right)\right)-\left(y_{x},\text{BM}\left(y_{x}\right)\right){||}_{2},\\ y_{x} & ≔\arg\underset{y\in S}{\min}||\left(x,\text{CSI}\left(x\right)\right)-\left(y,\text{BM}\left(y\right)\right){||}_{2}. \end{array} \end{array} \end{array}$$

Using this minimum distance method, we can obtain the thickness results by our proposed method. Next, we would like to present some measurements to compare our results with those by other methods.

### 3.3. Average Error and Maximal Error

In order to evaluate the thickness results, the average error and maximal error in an image are calculated to compare. The average error *err*_1_ is calculated by

(13) $$\begin{array}{c} \begin{array}{r} \text{er}{\text{r}}_{1}=\frac{1}{W}\overset{W}{\underset{i=1}{\sum }}\;|\;l\left(x_{i}\right)-l_{\text{truth}}\left(x_{i}\right)\;|\; \end{array}, \end{array}$$

where *x*_*i*_ = *i* for *i* = 1, ⋯, *W* represents the horizontal coordinate in each image, *l*(*x*) is the thickness function defined in (12) by our proposed method, *l*_truth_(*x*) is the thickness function provided by the ophthalmologists, and *W* is the image width (*W* = 600 in this paper). The maximal error err_2_ is

(14) $$\begin{array}{c} \begin{array}{r} \text{er}{\text{r}}_{2}=\underset{i\in \left\{1,2\cdots ,W\right\}}{\max}\;|\;l\left(x_{i}\right)-l_{\text{truth}}\left(x_{i}\right)\;|\; \end{array}. \end{array}$$

### 3.4. Dice Coefficient

Dice coefficient, a metric function in set comparing, is considered to measure the similarity between the segmentation result of the proposed method and the ground truth. It is defined as

(15) $$\begin{array}{c} \begin{array}{r} D=\frac{2\;|\;S_{\text{pro}}\cap S_{\text{truth}}\;|\;}{\;|\;S_{\text{pro}}\;|\;+\;|\;S_{\text{truth}}\;|\;} \end{array}, \end{array}$$

where sets *S*_*pro*_ and *S*_*truth*_ consist of the pixels in the segmented choroidal region by our proposed method and the manually labeled choroidal region by experts, respectively. ∣*S*∣ denotes the number of elements in the set *S*.

## 4. Discussion

**Figure 10:**
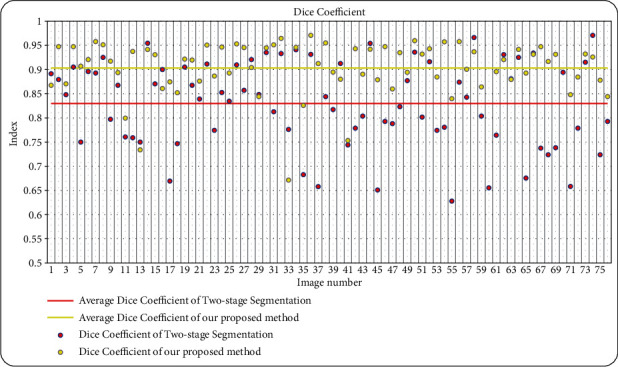
Dice coefficient comparison of all 76 test image results.

The details of the comparison in err_1_, err_2_, and Dice coefficient over the whole testing set (76 images) are given in Table 2. The two-stage segmentation is the method proposed in [24] which is verified that it performs better than some classical approaches such as Graph cut [41], *k*-means [41], and Graph Search Theory [26]. From Table 2, we can find that our proposed method possesses a smaller average error and larger Dice coefficient than the two-stage segmentation, which implies the effectiveness of the *l*_2_-*l*_*q*_ regression model and other improvements in our method. The comparison in Dice coefficient is visually shown in Figure 10. Yellow and red points represent the average Dice coefficient of our proposed method and the two-stage segmentation, respectively. It is clearly displayed that the Dice coefficient data obtained by our proposed method is generally superior to the data generated by the two-stage segmentation.

## 5. Conclusion

In this paper, we propose and implement an automated segmentation method based on CNN classifier and *l*_2_-*l*_*q*_ fitter to detect the region of the choroid. The BM, next to the inner surface of the choroid, is segmented by its physiological characteristic with the recognition of RPE. The extraction of the Choroidal-Scleral interface curve, outer surface of the choroid, is divided into two steps. First, we cut the images into small patches with label “on-line” or “off-line” to train the CNN classifier. The Focal Loss function and ADAM optimizer are used in the process of training the CNN model. Then, a binary classification problem is solved by using this CNN model with input test images. After obtaining the classified data with predicted values, we filter the mistake and noise points by the RANSAC method. In the second step, we adopt a *l*_2_-*l*_*q*_ (0 < *q* < 1) regression model to fit the discrete and filtered points to generate the desired curve. The hybrid OMP-SG algorithm is employed to solve the *l*_2_-*l*_*q*_ minimization. Segmentation results in some test images are given in Figure 7. Finally, we discuss and evaluate the experimental results in the aspects of average error, maximal error, and Dice coefficient. Comparison details with other methods are shown in Table 2 illustrating the effectiveness of the proposed method. With the help of the proposed method in autosegmentation of choroid from OCT images, the changes of choroidal thickness in response to experimental treatment or diseases could be effectively and accurately evaluated.

## Figures and Tables

**Algorithm 1:**
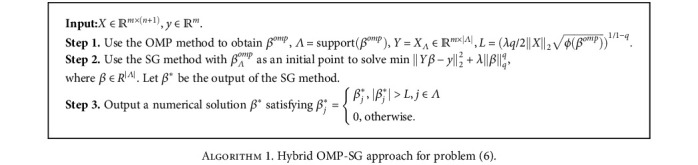
Hybrid OMP-SG approach for problem (6).

**Table 1:** Structure of CNN model used in this paper.

	Type	Filter size	Stride	Filter number	Padding
Layer 1	Convolution	3 × 3 × 1	1 × 1	128	1
Layer 2	ReLU	—	—	—	—
Layer 3	LRN	—	—	—	—
Layer 4	Convolution	3 × 3 × 128	1 × 1	64	1
Layer 5	ReLU	—	—	—	—
Layer 6	LRN	—	—	—	—
Layer 7	Convolution	3 × 3 × 64	1 × 1	32	1
Layer 8	ReLU	—	—	—	—
Layer 9	LRN	—	—	—	—
Layer 10	Fully connected	32 × 32 × 32	—	4096	—
Layer 11	ReLU	—	—	—	—
Layer 12	Fully connected	1 × 1 × 4096	—	1024	—
Layer 13	ReLU	—	—	—	—
Layer 14	Fully connected	1 × 1 × 1024	—	2	—
Layer 15	Softmaxloss	—	—	—	—

**Table 2:** Comparison in *err*_1_, *err*_2_, and Dice coefficient.

Method	Average error		Maximum error	Dice coefficient	
	Average	Standard deviation	Average	Average	Standard deviation
Proposed method	**2.0673**	**1.2348**	**7.1580**	**0.9035**	**0.0547**
Two-stage segmentation [24]	3.8239	2.0488	8.7801	0.8294	0.0893

## Data Availability

The data used to support the findings of this study were supplied by Optometry Research Clinic, School of Optometry, The Hong Kong Polytechnic University, under license and so cannot be made freely available. Requests for access to these data should be made to the Optometry Clinic in the Hong Kong Polytechnic University (Rachel Ka Man Chun, rachel.chun@polyu.edu.hk).
